# Health-related quality of life and its association with socioeconomic status and mental health in 5- to 7-year-old children: a cross-sectional study

**DOI:** 10.1007/s11136-024-03834-6

**Published:** 2024-11-20

**Authors:** Eva-Grethe Befus, Eirin Mølland, Sølvi Helseth, Thomas Westergren, Eirik Abildsnes, Milada Hagen, Sandra Nolte, Kristin Haraldstad

**Affiliations:** 1 Faculty of Health and Sport Sciences, University of Agder, P.O. Box 422, 4604 Kristiansand, Norway; 2 Faculty of Health Sciences, Oslo Metropolitan University, St. Olavs Plass, P.O. Box 4, 0130 Oslo, Norway; 3 Department of Economics and Finance, School of Business and Law, University of Agder, P.O. Box 422, 4604 Kristiansand, Norway; 4 Department of Public Health, University of Stavanger, P.O. Box 8600, 4036 Stavanger, Norway; 5 Institute of Health and Society, University of Oslo, Blindern, P.O. Box 1130, 0318 Oslo, Norway; 6 Person-Centred Research, Eastern Health Clinical School, Monash University, Melbourne, VIC Australia; 7 School of Health Sciences, Swinburne University of Technology, Melbourne, VIC Australia; 8 Pediatric Unit, Sørlandet Hospital Kristiansand, Lundsiden, P.O. Box 416, 4604 Kristiansand S, Norway

**Keywords:** (4–6) Health-related quality of life, Mental health, Children, Socioeconomic status, KIDSCREEN-27 proxy version, Strength and difficulties questionnaire proxy version

## Abstract

**Purpose:**

Assessing socioeconomic status (SES), mental health, and health-related quality of life (HRQoL) in young children is crucial for making informed health care decisions and identifying areas of intervention. The present study aimed to investigate potential associations between SES, mental health, and HRQoL in 5–7-year-old children.

**Method:**

The present study included mother-reported health assessments for 621 children aged 5–7 years in Grade 1 collected between 2019 and 2023 as part of the *Starting Right™* project. Online questionnaires were used to support public health nurses in assessing children’s health status. HRQoL (KIDSCREEN-27, 5 subscales) and mental health [Strength and Difficulties Questionnaire (SDQ), 4 subscales] were assessed. Sociodemographic characteristics, sex, maternal education, and income were obtained from Statistics Norway. The data were analyzed using multiple robust regression.

**Results:**

Mother-reported mean scores for the KIDSCREEN-27 were within the normal range compared with European norms (8–11 years). However, for each KIDSCREEN-27 dimension, there were individuals whose mothers reported scores that were substantially lower than average. Having mental health problems, defined as being in the 80th and 90th percentiles of the SDQ Total problem score, was associated with 2.1–10.7-point lower KIDSCREEN-27 scores (*p* < 0.001–0.021), which was most noticeable in the KIDSCREEN-27 school environment subscale. Weak but significant positive associations were found between SES and HRQoL.

**Conclusion:**

Our results provide important insights into the associations between SES, mental health, and HRQoL in young children. Given the strong association between mental health problems and HRQoL in Grade 1 children, the assessment of both is essential, so that early interventions, an improved caring environment, and nurturing support can be initiated.

**Supplementary Information:**

The online version contains supplementary material available at 10.1007/s11136-024-03834-6.

## Introduction

The World Health Organization (WHO) emphasizes that child health and well-being play a crucial role in fostering more sustainable societies [1]. Assessing health-related quality of life (HRQoL) in children is important because it provides a comprehensive understanding of a child’s subjective perspective on their physical, emotional, social, and functional well-being [2]. Furthermore, HRQoL assessment is essential for making informed health care decisions and identifying areas for intervention. HRQoL has increasingly become a central outcome in a variety of settings, including in public health research among children and adolescents [3]. However, there is limited knowledge about HRQoL among children younger than 7 years old [3]. Children in the general population tend to report high HRQoL. However, studies have also shown variations in scores, with some children reporting low HRQoL [4, 5]. This variability suggests that children reporting these particularly low scores should ideally be identified early for appropriate interventions. The transition from kindergarten can be a challenging experience [6], potentially causing mental health problems that need to be addressed and prevented [7]. Therefore, it seems especially important to understand HRQoL in younger children as they enter elementary school. HRQoL should, ideally, be measured by self-reported instruments [8]. However, when the child is too young, possibly lacking the cognitive skills to complete a questionnaire, proxy reports are recommended [9, 10].

Socioeconomic inequalities have a significant impact on families’ and children’s lives [11–13]. Children in families with low socioeconomic status (SES) may be deprived in various ways; experience tremendous stressors related to finances, education, and social participation; and be more likely to develop mental health problems [13–16]. Moreover, studies have shown that low SES is associated with lower HRQoL in children (aged 3–18 years) [11, 17, 18]. A low HRQoL, in turn, is associated with more mental health problems among children (aged 10–11 years) [19].

In fact, mental health problems in children cause major health impairments in HRQoL [20–24] and are regarded as one of the most serious health concerns of our time [25–28]. Symptoms of poor mental health can be detected as early as infancy and have been shown to follow individual and family trajectories throughout childhood and adolescence into adulthood [29, 30].

In a recent systematic review of 11 high-income countries, the prevalence of any childhood (ages 4–18 years) mental disorder was 12.7%, of which more than half did not receive treatment for their condition [31]. A Norwegian study reported a 7% prevalence of child mental disorders among 7–9-year-olds [32]. However, the distinction between parent-reported symptoms of poor mental health and clinically diagnosed mental disorders may not be clear [33]. A Danish population-based study on children aged 5–7 years revealed that symptoms of conduct problems were the most observed symptoms, followed by symptoms of emotional problems and hyperactivity-inattention, as measured with the Strengths and Difficulties Questionnaire (SDQ). Furthermore, a study showed that boys had a greater risk of having at least one mental health problem than girls [34]. Following the COVID-19 lockdown, in a German study, children aged 3–10 years old experienced more symptoms of hyperactivity and conduct problems than older children [35].

To our knowledge, a population-based study on the relationship between HRQoL, SES, and mental health problems in elementary school Grade 1 children has not yet been conducted, and earlier research has produced little direct evidence on HRQoL in 5–7-year-olds. Therefore, the main aim of the current study was to investigate the possible associations between SES and HRQoL, controlling for possible confounders, such as sex and age. Second, we aimed to investigate possible associations between mental health and HRQoL.

## Methods

### Study design

The current research is a substudy of the Norwegian *Starting Right™* project, which uses online proxy- and child-reported questionnaires to support public health nurses (PHNs) in assessing children’s health and development in routine follow-up [36]. The cross-sectional data were collected from students in Grade 1 in Agder County in southern Norway, who were recruited from both public and private schools. All Grade 1 students accompanied by parents were offered free consultation with the PHN. The mothers of the participating children answered online questionnaires concerning their child’s health prior to the consultation.

### Data collection

Data were collected from 2019 to 2023 using the SDQ for the assessment of mental health [37] and the KIDSCREEN-27 for the assessment of HRQoL [38]. Self-reports are the preferred method for assessing HRQoL because they allow individuals to directly provide information about their own health. However, in younger children, proxy reporting is recommended [8, 9, 39]. Prior to the individual consultation with the PHN at school, the proxy-reported online questionnaires were distributed to all Grade 1 parents as part of a start-of-school follow-up routine. All questionnaires were answered online prior to the consultation. Twentynine questionnaires were distributed before the child started Grade 1, as one of the municipalities altered their routines early in the project. With informed consent, the clinical data were aligned with Norwegian statistics and made available for the present study. Using unique personally identifiable IDs, data from the questionnaires were linked with administrative data from Statistics Norway, including maternal household income, educational level, and child sex. The parental role and child age data were obtained from *Starting Right™*.

## Instruments

### Health-related quality of life (KIDSCREEN-27)

To evaluate HRQoL, we applied the proxy version of the KIDSCREEN-27 questionnaire, a multidimensional measure of generic HRQoL consisting of 27 items grouped into five subscales: (1) physical well-being; (2) psychological well-being; (3) autonomy and parent relations; (4) social support and peers; and (5) school environment [40]. Previous research has provided support for its psychometric performance [41, 42]. The proxy version includes items similar to those in the child version, but it also includes questions about parents’ thoughts about their child’s feelings [40]. Item are assessed using a 5-point Likert scale, measuring either the strength of an attitude or the frequency of a behavior or emotion [40]. In line with the KIDSCREEN handbook [40], the Rasch scores were computed and transformed into T-scores with a general population normative mean of 50 and a standard deviation (SD) of 10. Higher scores indicate better HRQoL. Negatively worded items were reversed according to the manual. The normative values were based on data from an international survey sample including children aged 8–11 years old from 12 European countries [40].

### Socioeconomic status

We collected two common *indicators for SES* in the current study, which were maternal household income and maternal education. Our data included household income from mothers’ residences, also including fathers’ income when they are living together. Household income after tax per consumption unit was calculated using the European Union equivalence scale, as measured in 2020 and 2021. Our data did not include the exact household income values but included the mothers’ relative ranks in the household income distribution relative to the population of Norway. Statistics Norway provides income percentile data for individuals > 16 years living in Norway, here as categorized by sex. Our data show maternal household income percentiles. The maternal household income variable was divided into five even categories of percentiles: 1 = 0th–19th percentile, 2 = 20th–39th percentile, 3 = 40th–59th percentile, 4 = 60th–79th percentile and 5 = 80th–100th percentile. Maternal education was measured as the highest level of education completed by 2021. *Low educational levels* included elementary school, high school, and education based on senior high school but were not considered university or college education. *A high educational level* included any degree from a college or a university.

### Mental health problems (SDQs)

The Strength and Difficulties Questionnaire was used to assess children’s mental health problems, as reported by their mothers. The SDQ has been translated into Norwegian and has been used in general population studies of Norwegian children, demonstrating appropriate psychometric properties [43]. We used the 20 SDQ difficulty items representing four problem scales, namely (1) emotional problems, (2) peer problems, (3) conduct problems, and (4) hyperactivity-inattention, forming a Total problem score [44]. The SDQ subscale for prosocial behavior was not used, as we investigated mental health problems. Higher scores indicate more severe problems. The response scale is “not true”, “somewhat true”, or “certainly true”, rated 0–2 for negatively worded items and rated inversely 2–0 for positively worded items. Scores were calculated for each subscale (range 0–10) and for the Total problem score (0–40). Norwegian norms and cutoffs for the SDQ have not been established [43]; therefore, we calculated the 80th and 90th percentiles for the cutoff values within our study population for the SDQ Total problem score, similar to how developers calculate cutoffs in normative populations [45].

### Statistical analyses

Descriptive analyses were conducted using IBM SPSS Statistics (version 28). STATA (Stata-Corp. 2019, Stat Statistical Software: Release 17. College Station, TX, USA) was used to fit the robust regression models. Descriptive statistics were calculated for all variables and are presented as the mean and SD for continuous variables and counts and percentages for categorical variables. Because the assumptions for linear regression were not met and the residuals were skewed, we used robust regression to model possible associations between the dependent variable (the KIDSCREEN-27 subscales) and the selected independent variables. Univariate robust regression was conducted to examine possible associations between SES and HRQoL (KIDSCREEN-27 subscales: physical well-being, psychological well-being, autonomy and parent relations, social support and peers, and school environment), and between SDQ and HRQoL. Second, multiple robust regression was performed separately for SES (maternal household income and education) and the SDQ Total problem score in relation to HRQoL subscales while controlling for age and sex. Robust regressions were fitted separately for each of the five KIDSCREEN-27 subscales. Additionally, multiple robust regression was conducted for each KIDSCREEN-27 subscale, with the four SDQ scale scores as the independent variables, again controlling for age and sex. The results are presented as regression coefficients with 95% confidence intervals (CIs). P values ≤ 0.05 were considered to indicate statistical significance. All analyses were considered exploratory, so no correction for multiple testing was performed.

Sensitivity analyses were performed by excluding participants with maternal household income in the 0th–19th percentile because nontaxable income sometimes does not indicate low income in general. Sensitivity analyses were also performed excluding children who had not yet started school because some children were included in the project prior to the start of school.

## Results

### Characteristics of the sample

In total, mothers of 621 children participated in the study, with a response rate of 78%. Just over half (51.4%) of the included children were girls. All participating children were in Grade 1, with an age range of 5.1–7.3 years. Our study sample represented all groups of SES, however, it had a small overrepresentation of mothers with university or college education (65.4%) compared with Norwegian women aged 30–39 years old (54.8% nationally, 52.7% in Agder County, where the study was conducted), including women without children. One-third (32.3%) of the mothers had completed high school as their highest level of education, while 7.7% had completed elementary school only. Almost 30% of mothers had a household income between the 40th and 59th percentiles, while 12.6% of mothers had a household income above the 80th percentile of the national household income percentiles (Table 1).

**Table 1:** Characteristics of the sample

		N (%)
Total sample		621
Gender	*Girls*	319 (51.4)
	*Boys*	302 (48.6)
Age (years) (min–max)		5.1–7.3
	*Median*	6.5
Education level^a^	*Low*	208 (33.5)
	*High*	406 (65.4)
Missing		7 (1.1)
Income^b^	0th–19th percentile	69 (11.1)
	20th–39th percentile	156 (25.1)
	40th–59th percentile	177 (28.5)
	60th–79th percentile	123 (19.8)
	80th–100th percentile	78 (12.6)
Missing		18 (2.9)

^a^Mothers’ education level, low = elementary school or high school education and educations that are based on upper secondary school, but which are not approved as university and college education, high = university or college education

^b^The table illustrates which percentile the total household income of the mothers included in our study belongs to

### Proxy-reported HRQoL and mental health

**Fig. 1:**
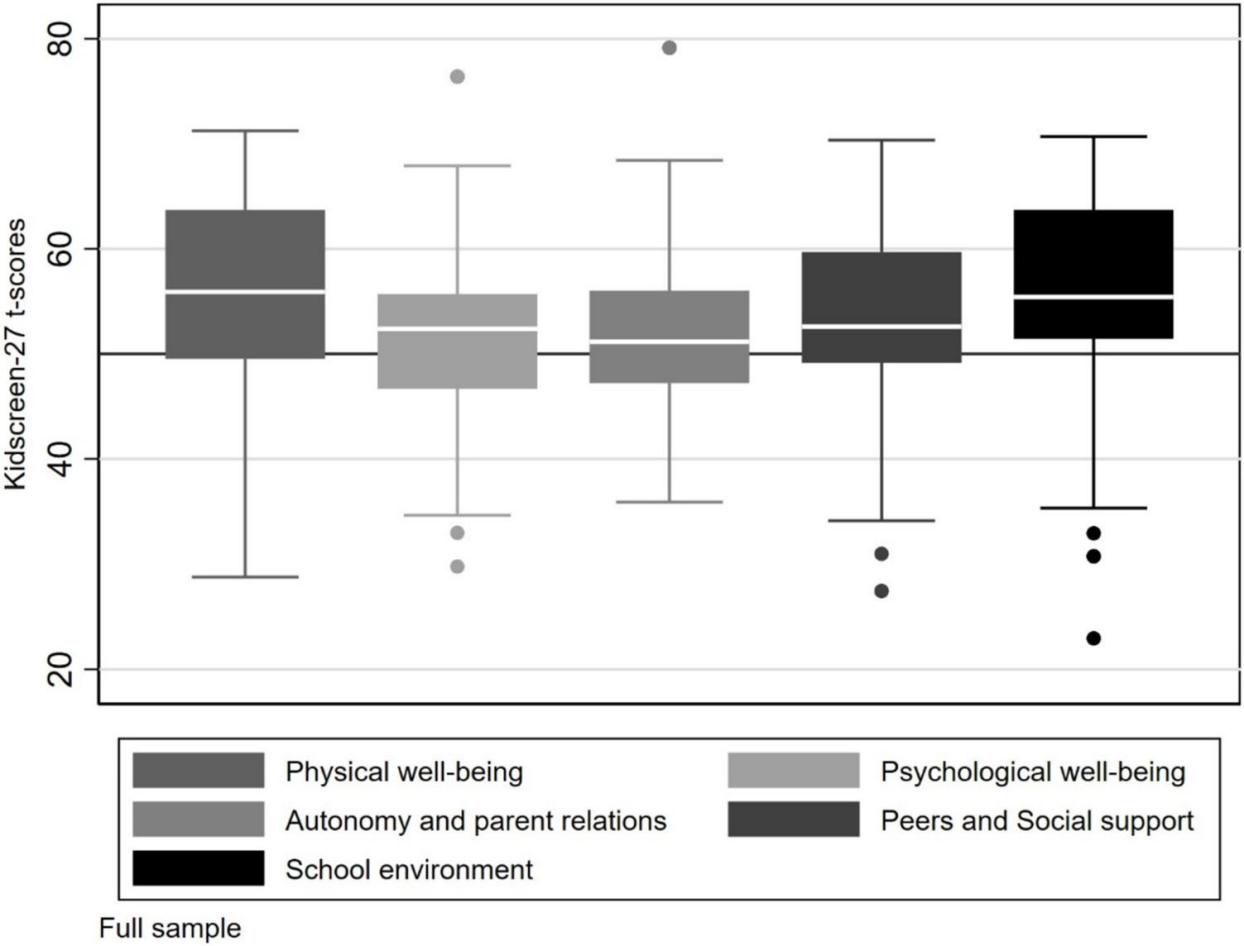
Boxplots representing the distribution of KIDSCREEN-27-scores in the full sample for each subscale. Box = 25th and 75th percentile, line = median, bars = min and max values

The mother-reported mean scores for all five KIDSCREEN-27 dimensions were above or well above the 50th percentile for the European KIDSCREEN-27 proxy report norm for children aged 8–11 years [40]. The distribution of each KIDSCREEN-27 subscale score is shown in Fig. 1. The mother reported mean KIDSCREEN-27 and SDQ scores are shown in Table 2. The internal consistency of the KIDSCREEN-27 questionnaire was confirmed, with a Cronbach’s alpha ranging from 0.70 to 0.83 for all subscales for the current sample. Values > 0.7 indicate acceptable reliability [46]. The Cronbach’s alpha [46] for the SDQ Total problem score in our sample was 0.78. The Cronbach’s alpha for the four problem scales ranged from 0.53–0.80, indicating an acceptable consistency for hyperactivity/inattention but low degree of internal consistency for emotional, peer, and conduct problems [46].

**Table 2:** Descriptive data of HRQoL (KIDSCREEN-27, parent version) and mental health (SDQ, parent version)

Instruments	Total (n = 621)	Cronbach’s alpha
KIDSCREEN-27^a^	Mean (SD)	
*Physical well-being*	55.9 (9.1)	0.78
Missing, n (%)	2 (0.3)	
*Psychological well-being*	52.2 (7.8)	0.77
Missing, n (%)	3 (0.4)	
*Autonomy and parent relations*	52.5 (7.6)	0.70
Missing, n (%)	23 (3.7)	
*Social support and peers*	53.2 (8.1)	0.82
Missing, n (%)	16 (2.6)	
*School environment*	57.0 (9.3)	0.83
Missing, n (%)	28 (4.5)	

Strength and Difficulties Questionnaire^b^	Mean (SD)	
*Total problem score (0–40)*	5.7 (4.3)	0.78
0–80th percentile^c^	474	
80th–90th percentile^c^	66	
90th–100th percentile^c^	63	
Missing, n (%)	18 (2.9)	
*Emotional symptoms (0–10)*	1.6 (1.7)	0.67
Missing, n (%)	18 (2.9)	
*Peer problems (0–10)*	0.7 (1.2)	0.55
Missing, n (%)	18 (2.9)	
*Conduct problems (0–10)*	1.0 (1.1)	0.53
Missing, n (%)	18 (2.9)	
*Hyperactivity/inattention (0–10)*	2.5 (2.2)	0.80
Missing, n (%)	18 (2.9)	

^a^For each KIDSCREEN-27 dimension, the Rasch scores were computed and transformed into T-scores with a general population mean of 50 and standard deviation (SD) of 10. Higher values indicate higher levels of HRQoL

^b^Mean scores for SDQ Total problem score (range 0–40) and 4 subdimensions (range 0–10). Higher values indicate more severe problems

^c^Based on the 80th and 90th percentiles from the present study from Agder County, Norway

### SES in association with HRQoL

When controlling for sex and age, having a mother with an income in the 80th–100th percentile was associated with 3.4 points greater scores for *physical well-being* (B = 3.4, 95% CI [0.1; 6.8]) and 3.8 points greater scores for the *school environment* subscale of KIDSCREEN-27 (B = 3.8; 95% CI [0.4; 7.2]) than having a mother with an income in the 0–19th percentile (Table 3). Having a mother with an income in the 60th–79th percentile was associated with a greater *autonomy and parent relations* score (B = 2.2, 95% CI [0.1; 4.3]) than having a mother with an income in the 0th–19th percentile. Being a boy was associated with lower subscale scores for *social support and peers* (− 1.7, 95% CI [− 3.1; − 0.4]) and *school environment* (− 2.5, 95% CI [− 4.1, − 0.9]). Furthermore, having a mother with a high educational level was associated with a higher subscale score for *school environment* (B = 2.3, 95% CI [0.5; 4.1]). In univariate analyses SES was positively associated with HRQoL (Table S1, supplementary materials).

**Table 3:** Robust multiple regression of SES in association with KIDSCREEN-27

KIDSCREEN-27 dimensions	Physical well-being		Psychological well-being		Autonomy and parent relation		Social support and peers		School environment	
	n = 595		n = 594		n = 573		n = 580		n = 568	
Predictor variable	B 95% CI	*p*-value	B 95% CI	*p*-value	B 95% CI	*p*-value	B 95% CI	*p*-value	B 95% CI	*p*-value
Boys (ref.: girls)	0.8	*0.323*	− 0.3	*0.615*	− 0.6	*0.260*	**−** **1.7**	***0.014***	**−** **2.5**	***0.003***
	− 0.8; 2.3		− 1.5; 0.9		− 1.7; 0.5		**−** **3.1; −** **0.4**		**−** **4.1; −** **0.9**	
Age (years)	− 0.6	*0.551*	**−** **2.5**	***0.003***	− 0.4	*0.597*	− 0.5	*0.561*	− 1.4	*0.268*
	− 2.8; 1.5		**−** **4.1; −** **0.8**		− 1.9; 1.1		− 2.4; 1.3		− 3.8; 1.1	
*Education level*^*a*^* (ref.: Low)*										
High	1.2	*0.162*	− 0.1	*0.901*	− 1.2	*0.063*	− 1.2	*0.120*	**2.3**	***0.014***
	− 0.5; 3.0		− 1.4; 1.3		− 2.4; 0.1		− 2.7; 0.3		**0.5; 4.1**	
Income^b^ (ref.: 0th–19th percentile)										
20th–39th percentile	1.0	*0.482*	2.0	*0.069*	0.9	*0.397*	1.3	*0.308*	2.3	*0.123*
	− 1.8; 3.8		− 0.2; 4.2		− 1.1; 2.9		− 1.2; 3.7		− 0.6; 5.2	
40th–59th percentile	0.7	*0.643*	0.4	*0.719*	1.6	*0.112*	0.6	*0.642*	1.2	*0.421*
	− 2.2; 3.5		− 1.8; 2.6		− 0.4; 3.6		− 1.9; 3.0		− 1.7; 4.1	
60th–79th percentile	2.0	*0.186*	0.9	*0.442*	**2.2**	***0.039***	1.9	*0.149*	2.1	*0.186*
	− 1.0; 5.0		− 1.4; 3.2		**0.1; 4.3**		− 0.7; 4.5		− 1.0; 5.2	
80th–100th percentile	**3.4**	***0.044***	2.5	*0.054*	1.7	*0.146*	1.7	*0.247*	**3.8**	***0.027***
	**0.1; 6.8**		− 0.0; 5.1		− 0.6; 4.1		− 1.2; 4.5		**0.4; 7.2**	

^a^Mother’s education level, low = elementary school, high school education or educations based on senior high school but which are not approved as university or college education, high = university or college education

^b^The percentiles were calculated based on all individuals aged > 16 years living in Norway. Household income was adjusted by the number of persons living in the household

Statistically significant values are shown in bold

Statistically significant *p*-values are shown in bold italics

### Mental health in association with HRQoL

In multiple robust regression analyses, the possible association between the SDQ Total problem score and HRQoL was investigated after adjusting for sex and age. As shown in Table 4, results indicated a significant association between mental health problems and lower HRQoL. Children scoring above the 90th percentile for the SDQ Total problem score had lower scores for KIDSCREEN-27 subscales *physical well-being* (B = − 4.3, 95% CI [− 6.8; − 1.8]), *social support and peers* (B = − 4.5, 95% CI [− 6.7; − 2.2]), *psychological well-being* (B = − 7.0, 95% CI [− 8.9; − 5.1]), and *school environment* (B = − 10.7, 95% CI [− 13.2; − 8.2]). Children classified between the 80th and 90th percentiles for the SDQ Total problem score showed 4.4 points lower on the KIDSCREEN-27 *school environment* subscale (B = − 4.4, 95% CI [− 6.8; − 2.0]) than children scoring below the 80th percentile for the SDQ. Finally, being a boy (B = − 1.9, 95% CI [− 3.4; − 0.4]) was associated with a lower HRQoL score in terms of *school environment* than being a girl.

**Table 4:** Robust multiple regression of SDQ total problem score in association with KIDSCREEN-27

KIDSCREEN-27 dimensions	Physical well-being		Psychological well-being		Autonomy and parent relation		Social support and peers		School environment	
	n = 602		n = 601		n = 580		n = 587		n = 575	
Predictor variable	B 95% CI	*p*-value	B 95% CI	*p*-value	B 95% CI	*p*-value	B 95% CI	*p*-value	B 95% CI	*p*-value
Boys (ref.: girls)	1.1	*0.175*	0.5	*0.433*	− 0.4	*0.475*	**−** **1.4**	***0.045***	**−** **1.9**	***0.014***
	− 0.5; 2.6		−0.7; 1.6		−1.5; 0.7		**−** **2.7; −** **0.0**		**−** **3.4; −** **0.4**	
Age (years)	− 0.2	*0.836*	**−2.2**	***0.005***	− 0.2	*0.801*	− 0.4	*0.653*	− 0.8	*0.515*
	− 2.3; 1.8		**−** **3.8; −** **0.7**		− 1.7; 1.3		− 2.2; 1.4		− 3.0; 1.5	
*SDQ total problem score (ref.: Normal)*										
80th–89th percentile^a^	− 2.1	*0.087*	**−** **3.1**	***0.001***	**−** **2.1**	***0.021***	− 2.0	*0.062*	**−** **4.4**	***< 0.001***
	− 4.6; 0.3		**−** **5.0; −** **1.3**		**−** **3.8; −** **0.3**		− 4.2; 0.1		**−** **6.8; −** **2.0**	
90th–100th percentile^a^	**−** **4.3**	***0.001***	**−** **7.0**	***< 0.001***	− 1.3	*0.173*	**−** **4.5**	***< 0.001***	**−** **10.7**	*< ****0.001***
	**−** **6.8; −** **1.8**		**−** **8.9; −** **5.1**		− 3.1; 0.5		**−** **6.7; −** **2.2**		**−** **13.2; −** **8.2**	

^a^Based on the 80th and 90th percentiles from the present study from Agder County, Norway

Statistically significant values are shown in bold

Statistically significant *p*-values are shown in bold italics

Multiple robust regression analyses, including the four SDQ subscales and controlling for sex and age, are shown in Table S3 in the supplementary materials. These analyses showed that having peer problems was associated with a significantly lower HRQoL for all KIDSCREEN-27 subscales. Furthermore, emotional symptoms, peer problems, conduct problems, and hyperactivity/inattention were all associated with lower KIDSCREEN-27 scores in school environment. Univariate analyses showed statistically significant associations between mental health problems and HRQoL (Table S2, supplementary materials).

To check whether there were significant differences in HRQoL measures pre and post the COVID-19 pandemic, we included a binary covariate pre versus post pandemic and reran all the multiple analyses with this new variable, as shown in supplementary materials Tables S4, S5 and S6. The COVID-19-variable was not statistically significant in any of the models.

### Sensitivity analyses

When we removed mothers with the lowest income, our results remained unchanged (data not shown). When children who had not started school were removed, our results also confirmed the main analyses (data not shown).

## Discussion

The present study bridges a knowledge gap on the association between SES, mental health, and proxy-reported HRQoL in 5–7-year-old children. We found a small positive, statistically significant association between maternal income/educational level and HRQoL on the physical well-being, autonomy and parent relations, and school environment KIDSCREEN-27 subscales. An important finding is the association between mental health problems and impaired HRQoL across all KIDSCREEN-27 subscales. Interestingly, compared with girls, boys had lower HRQoL scores for social support and peers, and school environment. The scores of the other three KIDSCREEN-27 subscales did not differ significantly between girls and boys.

Our findings indicated that low SES negatively impacts young children’s HRQoL, as was also shown in previous studies among children older than 6 years old [17, 18, 47, 48]. Our findings are supported by an Iranian study among 6–18-year-old students where high SES was associated with high school functioning [11]. However, this study used the Pediatric Quality of Life InventoryTM to measure HRQoL and the school domain may differ from KIDSCREEN-27. In our younger sample, the effect sizes of the associations between maternal household income and educational level and the abovementioned HRQoL subscales were small but significant. This is interesting and important because the SES of a child’s family is associated with HRQoL only to a small extent. The Norwegian welfare system includes tax-financed health and welfare services, and free education, which may have a mitigating effect on the impact of socioeconomic status on children’s HRQoL [49]. These findings suggest that a child’s socioeconomic background may be associated with poor health outcomes. However, subjective/proxy health parameters, such as the SDQ and KIDSCREEN-27 scores, add information for health dialog and attention to the child’s/parent’s experiences. This comprehensive approach enables agency for parents with children facing health challenges across the whole spectrum of SES. These parameters may be used to identify children at further risk of developing mental health problems and impaired HRQoL and to prevent such impairments by resourcing the caring environment of the child [50, 51]. Thus, the current study represents an important contribution to this field of research.

In the present sample, having mental health problems was strongly associated with impaired HRQoL, particularly for the school environment subscale KIDSCREEN-27. This important finding is supported by previous research [20–24]. However, these studies include self-reported data in the age range of 7–18 years old, whereas proxy reports regarding young children have not been studied. Thus, our findings provide new knowledge about young children at risk of poor HRQoL and its strong covariation with symptoms of poor mental health, which has rarely been investigated before. Importantly, in the *Starting Right™* project, the parent-reported KIDSCREEN-27 and SDQ instruments were used by the PHNs to understand and support the children with most problems, representing an approach different from categorizing the children in terms of psychopathology. Each child was offered a consultation with the PHN at school, where the specific needs of the child could be addressed [36]. The questionnaires were implemented in the already established school health services and may thereby have contributed to earlier identification of child problems, serving as a structured tool for dialog concerning the child’s health [36]. One point to consider when interpreting the questionnaire results is the fact that the questionnaires may have been answered with the purpose of influencing what could become a topic when the child visits the PHN. How well the parents knew and trusted the PHN may have affected their responses. Moreover, previous studies have shown that mothers report higher SDQ problem scores than fathers [43]. In this study we included solely maternal reports, which could have influenced our findings.

The mean mother-reported SDQ Total problem scores observed in the current study were comparable to those reported in previous studies conducted in Norway [43], Denmark [34] and Nordic countries [52]. We found a strong association between mental health problems and lower HRQoL, most noticeable in terms of school environment, followed by the dimensions of psychological well-being and social support and peers. Interestingly, all SDQ difficulties subscales were significantly associated with a reduced school environment KIDSCREEN-27 score. This may imply that children’s mental health is associated with how much they enjoy themselves at school, whether they can keep up at school, how they can pay attention, and whether they get along well with their teachers. However, we did not include the prosocial score from the SDQ in the current study, which could have added valuable information concerning relationships between positive mental health and HRQoL. Additionally, mental health problems and positive mental health are not necessarily correlated [53]. Previous research has shown that students (aged 10–14 years old) with good mental health feel more connected to school, are more receptive to teachers and school, achieve better academic performance, and are less involved in bullying than those in more vulnerable groups [54]. A study among 9–14-year-olds revealed an association between mental problems and low scores on the school environment KIDSCREEN-27 subscale, both self- and proxy-reported [55]. By including younger children, this study represents a supplement to this field of research, showing that mental health problems are strongly associated with the school environment subscale, even among school starters.

The current study reports proxy reports of HRQoL. In the 5–7-year-old age range, it is crucial to rely on proxy reports because of developmental limitations in cognitive abilities or cognitive skills in children [9]. Furthermore, proxy data may be used alongside self-reports, allowing for a comparison of the child’s own perception of their HRQoL with their parents’ or caregivers’ perspectives. This can help identify discrepancies and areas in which intervention may be needed [9]. Studies have shown that compliance between children and proxy respondents is often weaker for psychosocial domains and stronger for physical domains [10, 56]. However, proxy reports offer unique knowledge about the child’s HRQoL as an important supplement to the child’s subjective voice, both at the populational level and in the dialog between the PHN and parents. Nevertheless, the development of self-reported HRQoL instruments in 5–7-year-old children is essential [9].

Our study showed that boys scored lower on HRQoL for the social support and peers, and the school environment subscales than girls. Even if the effect size of the KIDSCREEN-27 subscales was small, this trend should still be noted. Previous research has shown that HRQoL deteriorates throughout childhood and adolescence, especially among girls [4, 41]. Our findings, among younger children, demonstrated that girls have greater HRQoL than boys for the aforementioned subscales. In addition, boys are commonly evaluated as having lower school readiness in preschool years than girls [57], which could explain the current findings of boys scoring lower on school environment HRQoL than girls. Furthermore, another Norwegian study among elementary school students (grades 1–10) revealed sex differences in terms of school well-being and school satisfaction. For boys, but not for girls, academic help from teachers was a strong predictive factor for good school well-being. For girls, however, loneliness was a predominant factor associated with an 80% reduced chance of reporting good school well-being [58]. Taken together, these findings call for an increased focus on and systematic assessment of HRQoL in children of all ages. It is crucial to capture HRQoL scores when the differences in HRQoL start to manifest themselves, so that appropriate interventions can be initiated. Moreover, interventions should be tailored to individuals mostly in need by the principle of proportionate universalism [59], which is in line with current national recommendations [60].

### Strengths and limitations

The major strengths of the present study are the relatively large sample size, the fact that participants were recruited from a variety of schools and that a high consent rate was achieved. Regarding the limitations of our study, we did not have information about the group that did not consent to participate. Our study included mothers’ responses only, which may differ from fathers’ reporting. Our study sample represented all groups of socioeconomic status; however, it had a small overrepresentation of mothers with higher education. One study recommended that the Norwegian proxy version of the KIDSCREEN-27 should be used with caution in 6-year-olds, particularly regarding psychological well-being and autonomy and parent relations [42]. Another limitation was the low Cronbach’s alpha for three of the SDQ subscales, which may have affected our findings. Furthermore, the present study included cross-sectional data only; hence, results should not be interpreted as causal. We did not intend to investigate the impact of COVID-19 on children’s HRQoL or mental health. However, restrictions during the pandemic have affected children’s health and well-being, especially in the least privileged families [61].Our SES variables are based on objective administrative data, which were the available variables in the project. However, use of subjectively reported scales could have added nuances and strengthened the study [62]. Regarding the association between SES and HRQoL, when controlling for possible confounders, maternal household income had a large CI [0.1; 6.8] [0.4; 7.2] (Table 3), which may indicate a large degree of heterogeneity in the observations obtained. The large CI suggests that the association between SES and HRQoL may vary in our sample. If we had a larger sample size, we could model interactions and possibly identify subgroups. The associations found between SES, mental health problems, and HRQoL may be influenced by other confounders that were not controlled for in the present study. We have only adjusted our multiple models for age and sex. Thus, we cannot rule out that the effect of both SES and SDQ on the outcome could be modified by such confounders. Further and more detailed analyses are just warranted.

## Conclusion

Our study addresses an important gap in knowledge concerning proxy-reported HRQoL and its association with SES and mental health in 5–7-year-old children. Overall, our study demonstrated that mental health problems in young children are strongly associated with impaired HRQoL, most noticeably in terms of school environment, followed by the dimensions of psychological well-being and social support and peers. Parental SES is weakly associated with a child’s HRQoL. Although the associations between SES and HRQoL are small, it is a concern that social inequality and its impact on HRQoL are noticeable in the young individuals. Early assessment of HRQoL, SES, and mental health in young children in the *Starting Right™* project is essential and provides important insight for parents, teachers, health professionals, and politicians. Hence, applied measures informed by reports of HRQoL and mental health assessments must be aimed at providing more nurturing support and an improved caring environment to those who need it the most as early as possible. The mental health, SES, and HRQoL of 5–7-year-olds in the general population have rarely been studied. These topics should be investigated further, along with PHN-initiated interventions to support those kids whose mothers report low HRQoL.

## Supplementary Information

Below is the link to the electronic supplementary material.

Supplementary file1 (DOCX 274 KB)

## Data Availability

The datasets generated and analysed during the current study are not publicly available due to regulation by the Norwegian Data Protection Authority but are available from the corresponding author on reasonable request.

## Abbreviations

B: Beta coefficient
CI: Confidence interval
HRQoL: Health-related quality of life
PHN: Public health nurse
SDQ: Strength and Difficulties Questionnaire
SES: Socioeconomic status
WHO: World Health Organization

## References

[1] Evans, D. (2017). *Investing in children: The European child and adolescent health strategy 2015–2020*. WHO Regional Office for Europe.

[2] Ravens-Sieberer, U., Erhart, M., Wille, N., Wetzel, R., Nickel, J., & Bullinger, M. (2006). Generic health-related quality-of-life assessment in children and adolescents: Methodological considerations. *PharmacoEconomics,**24*(12), 1199–1220. 10.2165/00019053-200624120-00005 17129075 10.2165/00019053-200624120-00005

[3] Befus, E.-G., Helseth, S., Mølland, E., Westergren, T., Fegran, L., & Haraldstad, K. (2023). Use of KIDSCREEN health-related quality of life instruments in the general population of children and adolescents: A scoping review. *Health and Quality of Life Outcomes,**21*(1), 6. 10.1186/s12955-023-02088-z 36670428 10.1186/s12955-023-02088-z PMC9857919

[4] Haraldstad, K., Christophersen, K.-A., & Helseth, S. (2017). Health-related quality of life and pain in children and adolescents: A school survey. *BMC Pediatrics,**17*(1), 174. 10.1186/s12887-017-0927-4 28738818 10.1186/s12887-017-0927-4 PMC5525195

[5] Michel, G., Bisegger, C., Fuhr, D. C., & Abel, T. (2009). Age and gender differences in health-related quality of life of children and adolescents in Europe: A multilevel analysis. *Quality of Life Research,**18*(9), 1147. 10.1007/s11136-009-9538-3.pdf 19774493 10.1007/s11136-009-9538-3

[6] Camović, D. (2023). Transition of children from kindergarten to elementary school–experiences of preschool teachers from Bosnia and Herzegovina. *Journal of Contemporary Educational Studies/Sodobna Pedagogika, 74*(1).

[7] Donaldson, C., Moore, G., & Hawkins, J. (2023). A systematic review of school transition interventions to improve mental health and wellbeing outcomes in children and young people. *School Mental Health,**15*(1), 19–35. 10.1007/s12310-022-09539-w

[8] Helseth, S., & Haraldstad, K. (2020). Child well-being. In F. Maggino (Ed.), *Encyclopedia of quality of life and well-being research* (pp. 1–5). Springer. 10.1007/978-3-319-69909-7_339-2

[9] Haverman, L., Limperg, P. F., Young, N. L., Grootenhuis, M. A., & Klaassen, R. J. (2017). Paediatric health-related quality of life: What is it and why should we measure it? *Archives of Disease in Childhood,**102*, 458–458. 10.1136/archdischild-2017-313105 27831905 10.1136/archdischild-2015-310068

[10] Khanna, D., Khadka, J., Mpundu-Kaambwa, C., Lay, K., Russo, R., Ratcliffe, J., Devlin, N., Norman, R., Viney, R., Ratcliffe, J., Dalziel, K., Mulhern, B., Hiscock, H., Street, D., Chen, G., Peasgood, T., Bailey, C., Mpundu-Kaambwa, C., Yu, A., … De Silva, A. (2022). Are we agreed? Self- versus proxy-reporting of paediatric health-related quality of life (HRQoL) using generic preference-based measures: A systematic review and meta-analysis. *PharmacoEconomics,**40*(11), 1043–1067. 10.1007/s40273-022-01177-z 35997957 10.1007/s40273-022-01177-z PMC9550745

[11] Hovsepian, S., Qorbani, M., Asadi, M., Hatami, M., Motlagh, M. E., Mahdavi-Gorabi, A., Noroozi, M., & Kelishadi, R. (2019). Socioeconomic inequalities in quality of life in Iranian children and adolescents: The weight disorder survey of the CASPIAN-IV study. *Journal of Research in Health Science, 19*(3), e00451. https://www.ncbi.nlm.nih.gov/pmc/articles/PMC7183559/pdf/jrhs-19-e00451.pdf PMC7183559 31586372

[12] You, Y., van Grieken, A., Estévez-López, F., Yang-Huang, J., & Raat, H. (2021). Factors associated with early elementary child health-related quality of life: The generation R study. *Frontiers in Public Health,**9*, 785054. 10.3389/fpubh.2021.785054 35155347 10.3389/fpubh.2021.785054 PMC8829330

[13] Francis, L., DePriest, K., Wilson, M., & Gross, D. (2018). Child poverty, toxic stress, and social determinants of health: Screening and care coordination. *Online Journal of Issues in Nursing,**23*(3), 2–2. 10.3912/OJIN.Vol23No03Man02 31427855 10.3912/OJIN.Vol23No03Man02 PMC6699621

[14] Reiss, F., Meyrose, A.-K., Otto, C., Lampert, T., Klasen, F., & Ravens-Sieberer, U. (2019). Socioeconomic status, stressful life situations and mental health problems in children and adolescents: Results of the German BELLA cohort-study. *PLoS ONE,**14*(3), e0213700. 10.1371/journal.pone.0213700 30865713 10.1371/journal.pone.0213700 PMC6415852

[15] Erhart, M., Ottova, V., Gaspar, T., Jericek, H., Schnohr, C., Alikasifoglu, M., Morgan, A., & Ravens-Sieberer, U. (2009). Measuring mental health and well-being of school-children in 15 European countries using the KIDSCREEN-10 Index. *International Journal of Public Health,**54*(Suppl 2), 160–166. 10.1007/s00038-009-5407-7 19652910 10.1007/s00038-009-5407-7

[16] Kinge, J. M., Øverland, S., Flatø, M., Dieleman, J., Røgeberg, O., Magnus, M. C., Evensen, M., Tesli, M., Skrondal, A., Stoltenberg, C., Vollset, S. E., Håberg, S., & Torvik, F. A. (2021). Parental income and mental disorders in children and adolescents: Prospective register-based study. *International Journal of Epidemiology,**50*(5), 1615–1627. 10.1093/ije/dyab066 33975355 10.1093/ije/dyab066 PMC8580274

[17] Costa, D., Cunha, M., Ferreira, C., Gama, A., Machado-Rodrigues, A. M., Rosado-Marques, V., Mendes, L. L., Nogueira, H., Pessoa, M., Silva, M. R. G., Velasquez-Melendez, G., & Padez, C. (2021). Socioeconomic inequalities in children’s health-related quality of life according to weight status. *American Journal of Human Biology*. 10.1002/ajhb.23453 32578372 10.1002/ajhb.23453

[18] Lapresa, L. B., Arizaleta, L. H., & Rajmil, L. (2012). Social inequalities in mental health and health-related quality of life in children in Spain. *Pediatrics,**130*(3), e528–e535. 10.1542/peds.2011-3594 22908114 10.1542/peds.2011-3594

[19] Wu, X., Veugelers, P. J., & Ohinmaa, A. (2021). Health behavior, health-related quality of life, and mental health among Canadian children: A population-based cohort study. *Frontiers in Nutrition,**8*(638259), 638259. 10.3389/fnut.2021.638259 33777992 10.3389/fnut.2021.638259 PMC7991792

[20] Rajmil, L., Palacio-Vieira, J. A., Herdman, M., López-Aguilà, S., Villalonga-Olives, E., Valderas, J. M., Espallargues, M., Alonso, J., Rajmil, L., Palacio-Vieira, J. A., Herdman, M., López-Aguilà, S., Villalonga-Olives, E., Valderas, J. M., Espallargues, M., & Alonso, J. (2009). Effect on health-related quality of life of changes in mental health in children and adolescents. *Health & Quality of Life Outcomes,**7*, 103–103. 10.1186/1477-7525-7-103 20030835 10.1186/1477-7525-7-103 PMC2805624

[21] Sharpe, H., Patalay, P., Fink, E., Vostanis, P., Deighton, J., & Wolpert, M. (2016). Exploring the relationship between quality of life and mental health problems in children: Implications for measurement and practice. *European Child & Adolescent Psychiatry,**25*(6), 659–667. 10.1007/s00787-015-0774-5 26498932 10.1007/s00787-015-0774-5

[22] Ravens-Sieberer, U., Wille, N., Erhart, M., Bettge, S., Wittchen, H.-U., Rothenberger, A., Herpertz-Dahlmann, B., Resch, F., Hölling, H., & Bullinger, M. (2008). Prevalence of mental health problems among children and adolescents in Germany: Results of the BELLA study within the National Health Interview and Examination Survey. *European Child & Adolescent Psychiatry,**17*, 22–33. 19132301 10.1007/s00787-008-1003-2

[23] Bettge, S., Wille, N., Barkmann, C., Schulte-Markwort, M., & Ravens-Sieberer, U. (2008). Depressive symptoms of children and adolescents in a German representative sample: Results of the BELLA study. *European Child & Adolescent Psychiatry,**17*(S1), 71–81. 10.1007/s00787-008-1008-x 19132306 10.1007/s00787-008-1008-x

[24] Ravens-Sieberer, U., & Kurth, B. (2008). The mental health module (BELLA study) within the German Health Interview and Examination Survey of Children and Adolescents (KiGGS): Study design and methods. *European Child & Adolescent Psychiatry,**17*, 10–21. 10.1007/s00787-008-1002-3 19132300 10.1007/s00787-008-1002-3

[25] Bor, W., Dean, A. J., Najman, J., & Hayatbakhsh, R. (2014). Are child and adolescent mental health problems increasing in the 21st century? A systematic review. *Australian & New Zealand Journal of Psychiatry,**48*(7), 606–616. 10.1177/0004867414533834 24829198 10.1177/0004867414533834

[26] Samji, H., Wu, J., Ladak, A., Vossen, C., Stewart, E., Dove, N., Long, D., & Snell, G. (2022). Review: Mental health impacts of the COVID-19 pandemic on children and youth—a systematic review. *Child and Adolescent Mental Health,**27*(2), 173–189. 10.1111/camh.12501 34455683 10.1111/camh.12501 PMC8653204

[27] World Health Organization. (2021). *Comprehensive mental health action plan 2013–2030.*

[28] World Health Organization. (2017). *Depression and other common mental disorders: global health estimates*.

[29] Nilsen, W., Kjeldsen, A., Karevold, E. B., Skipstein, A., Helland, M. S., Gustavson, K., Enstad, F., Baardstu, S., Røysamb, E., von Soest, T., & Mathiesen, K. S. (2017). Cohort profile: The tracking opportunities and problems study (TOPP)—study of Norwegian children and their parents followed from infancy to early adulthood. *International Journal of Epidemiology,**46*(5), 1399–1399g. 10.1093/ije/dyx057 28498979 10.1093/ije/dyx057

[30] Steinvoord, K., & Junge, A. (2022). Does an association exist between socio-economic status and subjective physical, mental and social well-being among early adolescents? *International Journal of Adolescent Medicine and Health*. 10.1515/ijamh-2019-0090 10.1515/ijamh-2019-0090 31586965

[31] Barican, J. L., Yung, D., Schwartz, C., Zheng, Y., Georgiades, K., & Waddell, C. (2022). Prevalence of childhood mental disorders in high-income countries: A systematic review and meta-analysis to inform policymaking. *Evidence Based Mental Health,**25*(1), 36–44. 10.1136/ebmental-2021-300277 34281985 10.1136/ebmental-2021-300277 PMC8788041

[32] Heiervang, E., Stormark, K. M., Lundervold, A. J., Heimann, M., Goodman, R., Posserud, M.-B., UllebØ, A. K., Plessen, K. J., Bjelland, I., Lie, S. A., & Gillberg, C. (2007). Psychiatric disorders in Norwegian 8- to 10-year-olds: An epidemiological survey of prevalence, risk factors, and service use. *Journal of the American Academy of Child and Adolescent Psychiatry,**46*(4), 438–447. 10.1097/chi.0b013e31803062bf 17420678 10.1097/chi.0b013e31803062bf

[33] Goodman, A., & Goodman, R. (2009). Strengths and difficulties Questionnaire as a dimensional measure of child mental health. *Journal of the American Academy of Child & Adolescent Psychiatry,**48*(4), 400–403. 10.1097/chi.0b013e3181985068 19242383 10.1097/CHI.0b013e3181985068

[34] Elberling, H., Linneberg, A., Olsen, E. M., Goodman, R., & Skovgaard, A. M. (2010). The prevalence of SDQ-measured mental health problems at age 5–7 years and identification of predictors from birth to preschool age in a Danish birth cohort: The Copenhagen Child Cohort 2000. *European Child and Adolescent Psychiatry,**19*, 725–735. 10.1007/s00787-010-0110-z 20419462 10.1007/s00787-010-0110-z

[35] Christner, N., Essler, S., Hazzam, A., & Paulus, M. (2021). Children’s psychological well-being and problem behavior during the COVID-19 pandemic: An online study during the lockdown period in Germany. *PLoS ONE,**16*(6), e0253473. 10.1371/journal.pone.0253473 34161376 10.1371/journal.pone.0253473 PMC8221463

[36] Westergren, T., Mølland, E., Haraldstad, K., Håland, Å. T., Koepp, U., Fegran, L., & Abildsnes, E. (2021). Implementation of the norwegian ‘Starting right’ child health service innovation: Implementation adjustments, adoption, and acceptability. *BMC Health Services Research*. 10.1186/s12913-021-06096-x 33485333 10.1186/s12913-021-06096-x PMC7824922

[37] Sveen, T. H., Berg-Nielsen, T. S., Lydersen, S., & Wichstrøm, L. (2013). Detecting psychiatric disorders in preschoolers: Screening with the Strengths and Difficulties Questionnaire. *Journal of the American Academy of Child and Adolescent Psychiatry,**52*(7), 728–736. 10.1016/j.jaac.2013.04.010 23800486 10.1016/j.jaac.2013.04.010

[38] Andersen, J. R., Natvig, G. K., Haraldstad, K., Skrede, T., Aadland, E., & Resaland, G. K. (2016). Psychometric properties of the Norwegian version of the Kidscreen-27 questionnaire. *Health and Quality of Life Outcomes,**14*(1), 58. 10.1186/s12955-016-0460-4 27062022 10.1186/s12955-016-0460-4 PMC4826483

[39] Ravens-Sieberer, U., Karow, A., Barthel, D., & Klasen, F. (2014). How to assess quality of life in child and adolescent psychiatry. *Dialogues in Clinical Neuroscience,**16*(2), 147–158. 10.31887/dcns.2014.16.2/usieberer 25152654 10.31887/DCNS.2014.16.2/usieberer PMC4140509

[40] Ravens-Sieberer, U. (2006). *The Kidscreen questionnaires: Quality of life questionnaires for children and adolescents; handbook*. Pabst Science Publ.

[41] Ravens-Sieberer, U., Auquier, P., Erhart, M., Gosch, A., Rajmil, L., Bruil, J., Power, M., Duer, W., Cloetta, B., & Czemy, L. (2007). The KIDSCREEN-27 quality of life measure for children and adolescents: psychometric results from a cross-cultural survey in 13 European countries. *Quality of Life Research,**16*(8), 1347–1356. 10.1007/s11136-007-9240-2.pdf 17668292 10.1007/s11136-007-9240-2

[42] Riiser, K., Helseth, S., Christophersen, K.-A., & Haraldstad, K. (2020). Confirmatory factor analysis of the proxy version of Kidscreen-27 and relationships between health-related quality of life dimensions and body mass index and physical activity in young schoolchildren. *Preventive Medicine Reports,**20*, 101210. 10.1016/j.pmedr.2020.101210 32995148 10.1016/j.pmedr.2020.101210 PMC7516181

[43] Kornør, H., & Heyerdahl, S. (2017). Måleegenskaper ved den norske versjonen av Strengths and Difficulties Questionnaire, foreldrerapport (SDQ-P).

[44] Goodman, R. (2001). Psychometric properties of the Strengths and Difficulties Questionnaire. *Journal of the American Academy of Child and Adolescent Psychiatry,**40*(11), 1337–1345. 10.1097/00004583-200111000-00015 11699809 10.1097/00004583-200111000-00015

[45] SDQinfo.org. (2016). *Scoring the SDQ.* Retrieved 20 April from https://www.sdqinfo.org/py/sdqinfo/c0.py

[46] Pallant, J. (2020). *SPSS survival manual: A step by step guide to data analysis using IBM SPSS* (7th ed.). Open University Press.

[47] Ravens-Sieberer, U., Kaman, A., Erhart, M., Devine, J., Schlack, R., & Otto, C. (2022). Impact of the COVID-19 pandemic on quality of life and mental health in children and adolescents in Germany. *European Child & Adolescent Psychiatry,**31*(6), 879–889. 10.1007/s00787-021-01726-5 33492480 10.1007/s00787-021-01726-5 PMC7829493

[48] Rajmil, L., Herdman, M., Ravens-Sieberer, U., Erhart, M., & Alonso, J. (2014). Socioeconomic inequalities in mental health and health-related quality of life (HRQOL) in children and adolescents from 11 European countries. *International Journal of Public Health,**59*(1), 95–105. 10.1007/s00038-013-0479-9 23793782 10.1007/s00038-013-0479-9

[49] OECD. (2022). *Education at a Glance 2022*. 10.1787/3197152b-en

[50] World Health Organization. (2023). Nurturing care framework progress report 2018–2023: Reflections and looking forward.

[51] Garner, A., & Yogman, M. (2021). Preventing Childhood toxic stress: Partnering with families and communities to promote relational health. *Pediatrics,**148*(2), e2021052582. 10.1542/peds.2021-052582 34312296 10.1542/peds.2021-052582

[52] Obel, C., Heiervang, E., Rodriguez, A., Heyerdahl, S., Smedje, H., Sourander, A., Guðmundsson, Ó.O., Clench-Aas, J., Christensen, E., Heian, F., Mathiesen, K., Magnússon, P. L., Njarðvík, U. U., Koskelainen, M., Rønning, J., Stormark, K., & Olsen, J. R. (2004). The Strengths and Difficulties Questionnaire in the Nordic countries. *European Child and Adolescent Psychiatry, 13*(S2). 10.1007/s00787-004-2006-2 10.1007/s00787-004-2006-2 15243784

[53] Holen, S., Lervåg, A., Waaktaar, T., & Ystgaard, M. (2012). Exploring the associations between coping patterns for everyday stressors and mental health in young schoolchildren. *Journal of School Psychology, 50*(2), 167–193. https://www.sciencedirect.com/science/article/pii/S0022440511000860?via%3Dihub 10.1016/j.jsp.2011.10.006 22386119

[54] Arslan, G., & Allen, K.-A. (2022). Complete mental health in elementary school children: Understanding youth school functioning and adjustment. *Current Psychology,**41*(3), 1174–1183. 10.1007/s12144-020-00628-0

[55] Dey, M., Mohler-Kuo, M., & Landolt, M. (2012). Health-related quality of life among children with mental health problems: A population-based approach. *Health and quality of life outcomes,**10*, 73. 10.1186/1477-7525-10-73 22709358 10.1186/1477-7525-10-73 PMC3420327

[56] Rajmil, L., López, A. R., López-Aguilà, S., & Alonso, J. (2013). Parent-child agreement on health-related quality of life (HRQOL): A longitudinal study. *Health & Quality of Life Outcomes,**11*(1), 101–101. 10.1186/1477-7525-11-101 23786901 10.1186/1477-7525-11-101 PMC3706362

[57] Flatø, M., Bratsberg, B., Kotsadam, A., Torvik, F. A., Røgeberg, O., & Stoltenberg, C. (2023). Ready for school? Effects on school starters of establishing school psychology offices in Norway.

[58] Løhre, A., Moksnes, U. K., & Lillefjell, M. (2014). Gender differences in predictors of school wellbeing? *Health Education Journal,**73*(1), 90–100. 10.1177/0017896912470822

[59] Carey, G., Crammond, B., & De Leeuw, E. (2015). Towards health equity: A framework for the application of proportionate universalism. *International Journal for Equity in Health*. 10.1186/s12939-015-0207-6 26369339 10.1186/s12939-015-0207-6 PMC4570091

[60] Helse- og omsorgsdepartementet. (2022–2023). *Meld. St. 15 (2022–2023) Fokehelsemeldinga, Nasjonal strategi for utjamning av sosiale helseforskjellar*.

[61] Nøkleby, H., Borge, T. C., Lidal, I. B., Johansen, T. B., & Langøien, L. J. (2023). Konsekvenser av covid-19-pandemien for barn og unges liv og psykiske helse: andre oppdatering av en hurtigoversikt [Consequences of the Covid-19 pandemic on children and youth’s life and mental health: Second update of a rapid review].

[62] Kim, K. W., Wallander, J. L., Peskin, M., Cuccaro, P., Elliott, M. N., & Schuster, M. A. (2018). Associations between parental SES and children’s health-related quality of life: The role of objective and subjective social status. *Journal of Pediatric Psychology,**43*(5), 534–542. 10.1093/jpepsy/jsx139 29155956 10.1093/jpepsy/jsx139

[63] World Medical Association. (2001). World Medical Association Declaration of Helsinki. Ethical principles for medical research involving human subjects. *Bulletin of the World Health Organization, 79*(4), 373. PMC2566407 11357217

[64] The General Data Protection Regulation [Personopplysningsloven], LOV-2018-06-15-38 (2018). https://lovdata.no/dokument/NL/lov/2018-0615-38

