# Supplementary material for: Health-related quality of life and its association with socioeconomic status and mental health in 5- to 7-year-old children: a cross-sectional study
Source: Qual Life Res. 2024 Nov 20;34(2):549–61. doi: 10.1007/s11136-024-03834-6 (PMC11865220; doi:10.1007/s11136-024-03834-6)
Supplement: Supplementary file 1 — Supplementary file1 (DOCX 274 KB) [file 11136_2024_3834_MOESM1_ESM.docx]

**Article title**

Health-related quality of life and its association with socioeconomic status and mental health in 5- to 7-year-old children – A cross-sectional study

**Journal title:** Quality of Life Research

**Author information**

Corresponding author:

Eva-Grethe Befus^1,8^, eva-grethe.befus@uia.no, ORCID-ID: 0000-0002-4577-8639

Coauthors:

Eirin Mølland^3^, eirin.molland@uia.no, ORCID-ID: 0000-0002-2403-936X

Sølvi Helseth^2,1^, solvi@oslomet.no, ORCID-ID: 0000-0002-6411-9057

Thomas Westergren^4,1^, per.c.westergren@uis.no, ORCID-ID: 0000-0003-4253-1996

Eirik Abildsnes^5^, [eirik.abildsnes@medisin.uio.no](mailto:eirik.abildsnes@medisin.uio.no), ORCID-ID: [0000-0002-5454-8560](https://orcid.org/0000-0002-5454-8560)

Milada Hagen^2,1^, milasm@oslomet.no, ORCID-ID: 0000-0001-8947-8649

Sandra Nolte^6,7^, [sandra.nolte@monash.edu](mailto:sandra.nolte@monash.edu), ORCID-ID: 0000-0001-6185-9423

Kristin Haraldstad^1^, kristin.haraldstad@uia.no, ORCID-ID: 0000-0002-7364-147X

^1^ Faculty of Health and Sport Sciences, University of Agder, P.O. Box 422, 4604 Kristiansand, Norway

^2^ Faculty of Health Sciences, Oslo Metropolitan University, St. Olavs plass, P.O. Box 4, 0130 Oslo, Norway

^3^ Department of Economics and Finance, School of Business and Law, University of Agder, P.O. Box 422, 4604 Kristiansand, Norway

^4^ Department of Public Health, University of Stavanger, P.O. Box 8600, 4036 Stavanger, Norway

^5^ Institute of Health and Society, University of Oslo, P.O. Box 1130 Blindern, 0318 Oslo, Norway

^6^ Person-Centered Research, Eastern Health Clinical School, Monash University, Melbourne, Victoria, Australia

^7^ School of Health Sciences, Swinburne University of Technology, Melbourne, Victoria, Australia

^8^ Pediatric Unit, Sørlandet Hospital Kristiansand, P.O. Box 416 Lundsiden, 4604 Kristiansand S, Norway

Supplementary tables available upon request:

| Table S1 Univariate, robust regression of SES in association with KIDSCREEN-27 | | | | | | | | | | |
| --- | --- | --- | --- | --- | --- | --- | --- | --- | --- | --- |
| Dimensions | **Physical**  **well-being** | | **Psychological well-being** | | **Autonomy and parent relation** | | **Social support and peers** | | **School environment** | |
|  | n=595 | | n=594 | | n=573 | | n=580 | | n=568 | |
| Predictor variable | B  95% CI | *p*-value | B  95% CI | *p*-value | B  95% CI | *p*-value | B  95% CI | *p*-value | B  95% CI | *p*-value |
| Education level^a^ (ref.: Low) |  |  |  |  |  |  |  |  |  |  |
| High | 1.2  -0.6; 2.9 | *.186* | -0.1  -1.5; 1.2 | *.857* | -1.1  -2.3; 0.1 | *.073* | -1.1  -2.6; 0.4 | *.160* | **2.5**  **0.7; 4.3** | ***.007*** |
| Income^b^ (ref.: 0–19th percentile) |  |  |  |  |  |  |  |  |  |  |
| 20–39th percentile | 0.9  -1.9; 3.7 | *.527* | 1.9  -0.3; 4.1 | *.088* | 0.8  -1.2; 2.8 | *.416* | 1.2  -1.3; 3.6 | *.340* | 2.1  -0.8; 5.1 | *.148* |
| 40–59th percentile | 0.6  -2.2; 3.4 | *.668* | 0.0  -2.2; 2.3 | *.967* | 1.5  -0.5; 3.5 | *.136* | 0.2  -2.2; 2.7 | *.856* | 0.8  -2.1; 3.7 | *.582* |
| 60–79th percentile | 1.8  -1.1; 4.8 | *.225* | 0.8  -1.5; 3.1 | *.502* | **2.2**  **0.1; 4.3** | ***.038*** | 1.9  -0.7; 4.5 | *.147* | 2.1  -1.0; 5,1 | *.193* |
| 80–100th percentile | 3.3  -0.0; 6.6 | *.051* | 2.1  -0.5; 4.6 | *.120* | 1.7  -0.7; 4.0 | *.158* | 1.5  -1.3; 4.4 | *.296* | **3.6**  **0.2; 7.0** | ***.035*** |
| ^a^Mother’s education level, low=elementary school, high school education or educations based on senior high school but which are not approved as university or college education, high=university or college education  ^b^The percentiles were calculated based on all individuals aged > 16 years living in Norway. Household income was adjusted by the number of persons living in the household. | | | | | | | | | | |

| Table S2 Univariate, robust regression of SDQ in association with KIDSCREEN-27 | | | | | | | | | | |
| --- | --- | --- | --- | --- | --- | --- | --- | --- | --- | --- |
| Dimensions | **Physical**  **well-being** | | **Psychological well-being** | | **Autonomy and parent relation** | | **Social support and peers** | | **School environment** | |
|  | n=602 | | n=601 | | n=580 | | n=587 | | n=575 | |
| Predictor variable | B  95% CI | *p*-value | B  95% CI | *p*-value | B  95% CI | *p*-value | B  95% CI | *p*-value | B  95% CI | *p*-value |
| SDQ |  |  |  |  |  |  |  |  |  |  |
| Emotional symptoms | **-1.3**  **-1.7; -0.8** | ***<.001*** | **-1.2**  **-1.5; -0.8** | ***<.001*** | **-0.4**  **-0.7; -0.1** | ***.032*** | -0.2  -0.6; 0.2 | *.334* | **-0.6**  **-1.0; -0.2** | ***.007*** |
| Peer problems | **-1.1**  **-1.8; -0.5** | ***.001*** | **-0.6**  **-1.1; -0.1** | ***.015*** | **0.6**  **0.1; 1.0** | ***.019*** | **-1.7**  **-2.3; -1.1** | ***<.001*** | **-1.2**  **-1.8; -0.5** | ***<.001*** |
| Conduct problems | 0.1  -0.6; 0.9 | *.691* | **-0.7**  **-1.3; -0.2** | ***.010*** | **-0.7**  **-1.2; -0.1** | ***.015*** | -0.3  -1.0; 0.3 | *.308* | **-1.4**  **-2.1; -0.7** | ***<.001*** |
| Hyperactivity-inattention | 0.2  -0.2; 0.5 | *.377* | -0.2  -0.5; 0.1 | *.109* | -0.2  -0.5; 0.1 | *.119* | -0.2  -0.6; -0.1 | *.177* | **-1.1**  **-1.4; -0.7** | ***<.001*** |

| Table S3 Robust multiple regression of SDQ subscales in association with KIDSCREEN-27 | | | | | | | | | | |
| --- | --- | --- | --- | --- | --- | --- | --- | --- | --- | --- |
| KIDSCREEN-27  dimensions | **Physical**  **well-being** | | **Psychological well-being** | | **Autonomy and parent relation** | | **Social support and peers** | | **School environment** | |
|  | n=602 | | n=601 | | n=580 | | n=587 | | n=575 | |
| Predictor Variable | B  95% CI | *p*-value | B  95% CI | *p*-value | B  95% CI | *p*-value | B  95% CI | *p*-value | B  95% CI | *p*-value |
| Boys (ref.: girls) | 0.8  -0.7; 2.3 | *.308* | 0.5  -0.6; 1.6 | *.374* | -0.2  -1.3; 0.9 | *.769* | **-1.5**  **-2.8; -0.2** | ***.022*** | **-1.5**  **-2.9; -0.0** | ***.047*** |
| Age (years) | -0.0  -2.0; 2.0 | *.989* | **-2.1**  **-3.6; -0.6** | ***.006*** | -0.1  -1.6; 1.4 | *.894* | -0.6  -2.4; 1.2 | *.503* | -0.9  -3.0; 1.3 | *.419* |
| SDQ |  |  |  |  |  |  |  |  |  |  |
| Emotional symptoms | **-1.3**  **-1.7; -0.8** | ***<.001*** | **-1.1**  **-1.5; -0.8** | ***<.001*** | **-0.4**  **-0.7; -0.0** | ***.033*** | -0.2  -0.6; 0.2 | *.335* | **-0.6**  **-1.0; -0.2** | ***.007*** |
| Peer problems | **-1.1**  **-1.8; -0.5** | ***.001*** | **-0.6**  **-1.1; -0.1** | ***.016*** | **0.6**  **0.1; 1.0** | ***.020*** | **-1.7**  **-2.3; -1.2** | ***<.001*** | **-1.2**  **-1.8; -0.6** | ***<.001*** |
| Conduct problems | 0.1  -0.6; 0.8 | *.778* | **-0.7**  **-1.3; -0.2** | ***.011*** | **-0.7**  **-1.2; -0.1** | ***.018*** | -0.2  -0.9; 0.4 | *.490* | **-1.3**  **-2.0; -0.6** | ***<.001*** |
| Hyperactivity-inattention | 0.1  -0.2; 0.5 | *.441* | -0.3  -0.6; 0.0 | *.050* | -0.2  -0.5; 0.1 | *.132* | -0.2  -0.5; 0.1 | *.264* | **-1.0**  **-1.4; -0.7** | ***<.001*** |


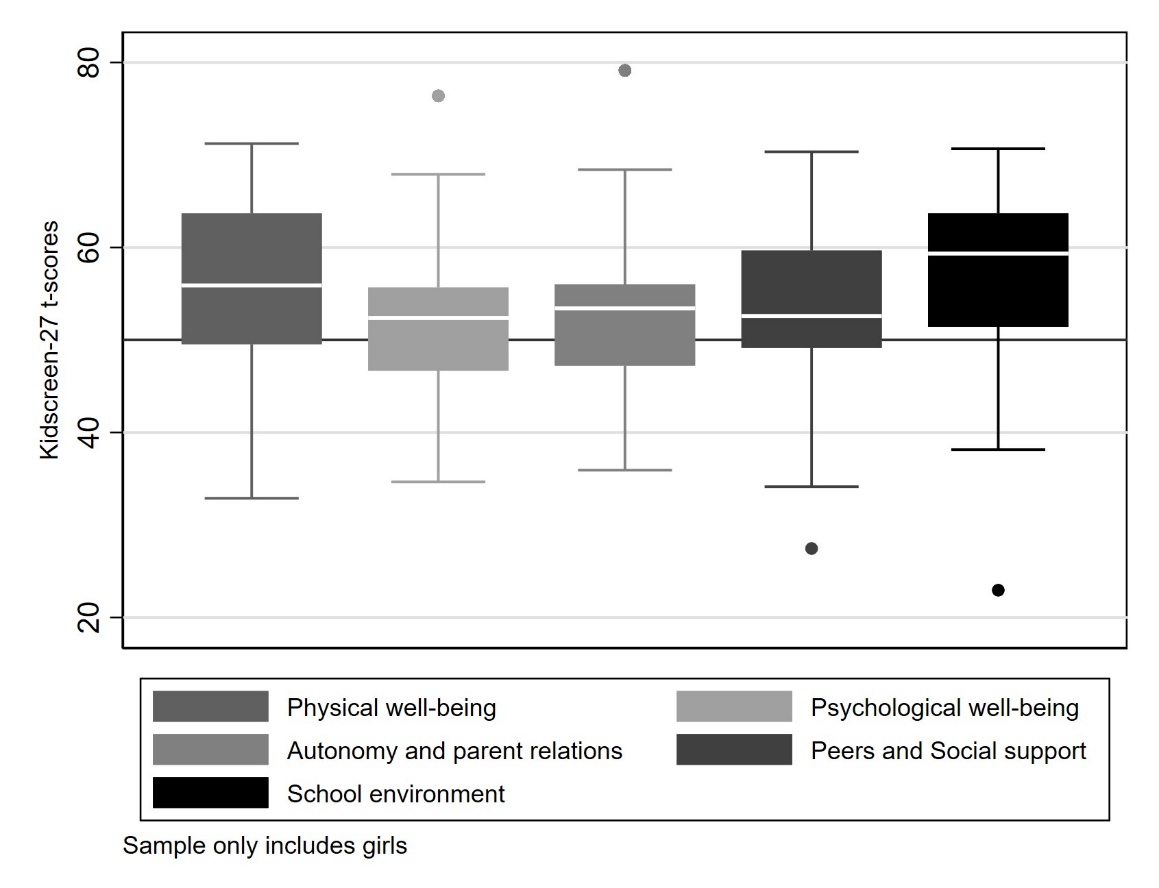


**Fig. 2** Boxplots representing the distribution of KIDSCREEN-27-scores in girls only for

each subscale. Box = 25th and 75th percentile, line = median, bars = min and max values


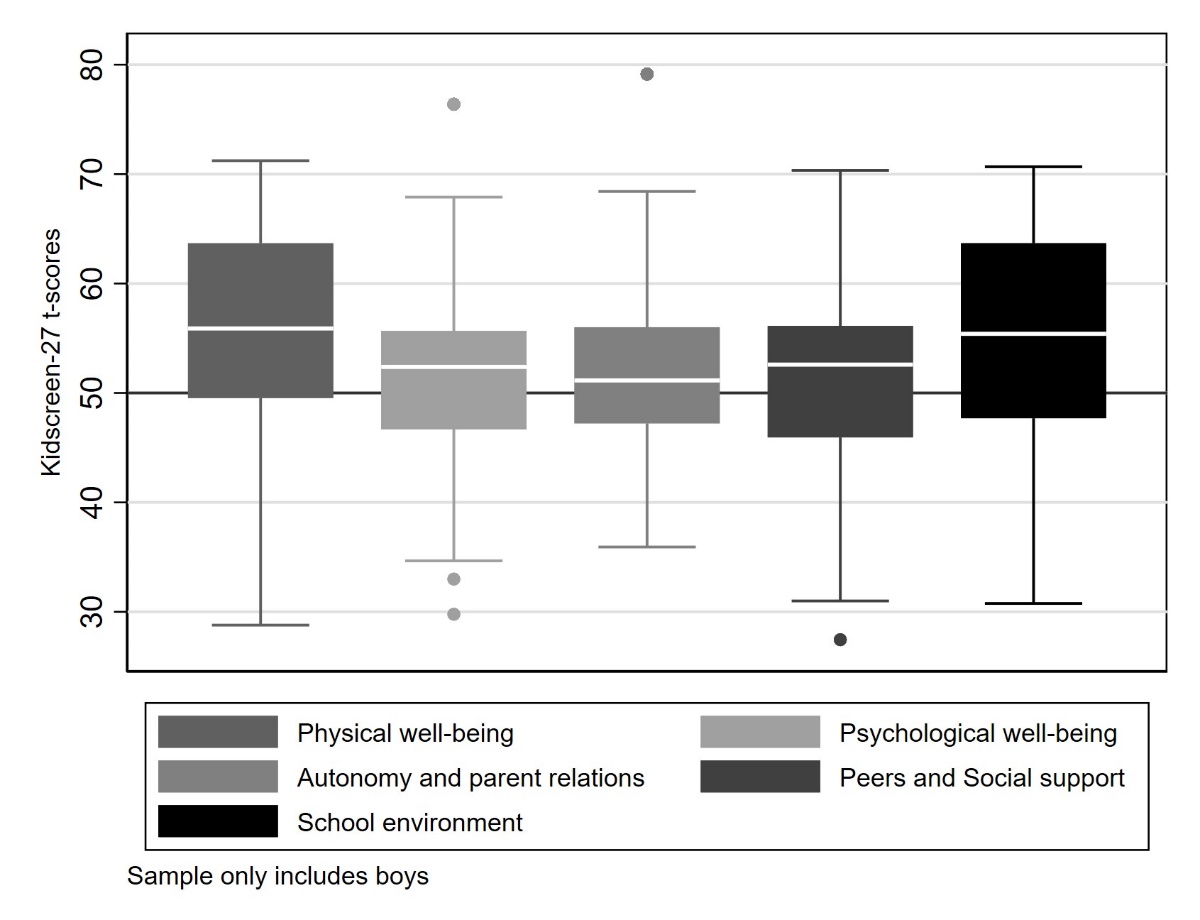


**Fig. 3** Boxplots representing the distribution of KIDSCREEN-27-scores in boys only, for

each subscale. Box = 25th and 75th percentile, line = median, bars = min and max values

| Table S4 Robust multiple regression of SES in association with KIDSCREEN-27, comparing children assessed pre (n= 143) versus post pandemic (n=478). | | | | | | | | | | |
| --- | --- | --- | --- | --- | --- | --- | --- | --- | --- | --- |
| Dimensions | **Physical**  **well-being** | | **Psychological well-being** | | **Autonomy and parent relation** | | **Social support and peers** | | **School environment** | |
|  | B  95% CI | *p*-value | B  95% CI | *p*-value | B  95% CI | *p*-value | B  95% CI | *p*-value | B  95% CI | *p*-value |
| Post pandemic* (ref. pre pandemic) | -1.0  -2.9; 0.9 | *.283* | 1.3  -1.; 2.7 | *.078* | 0.3  -1.0; 1.6 | *.642* | 1.0  -0.7; 2.6 | *.238* | 1.0  -1.0; 3.0 | *.321* |
| *Controlling for maternal household income, maternal education, age and sex | | | | | | | | | | |

| Table S5 Robust multiple regression of total SDQ in association with KIDSCREEN-27, comparing children assessed pre (n= 143) versus post pandemic (n=478). | | | | | | | | | | |
| --- | --- | --- | --- | --- | --- | --- | --- | --- | --- | --- |
| Dimensions | **Physical**  **well-being** | | **Psychological well-being** | | **Autonomy and parent relation** | | **Social support and peers** | | **School environment** | |
|  | B  95% CI | *p*-value | B  95% CI | *p*-value | B  95% CI | *p*-value | B  95% CI | *p*-value | B  95% CI | *p*-value |
| Post pandemic* (ref. pre pandemic) | -1.6  -3.4; 0.3 | *.092* | 1.1  -0.3; 2.5 | *.115* | 0.3  -1.0; 1.6 | *.603* | 1.1  -0.5; 2.7 | *.167* | 0.2  -1.7; 2.0 | *.859* |
| *Controlling for total SDQ problem score, age and sex | | | | | | | | | | |

| Table S6 Robust multiple regression of SES, 4 SDQ problem subscales in association with KIDSCREEN-27, comparing children assessed pre (n= 143) versus post pandemic (n=478). | | | | | | | | | | |
| --- | --- | --- | --- | --- | --- | --- | --- | --- | --- | --- |
| Dimensions | Physical  well-being | | Psychological well-being | | Autonomy and parent relation | | Social support and peers | | School environment | |
|  | B  95% CI | *p*-value | B  95% CI | *p*-value | B  95% CI | *p*-value | B  95% CI | *p*-value | B  95% CI | *p*-value |
| Post pandemic* (ref. pre pandemic) | -1.1  -2.9; 0.8 | *.254* | 0.8  -0.6; 2.1 | *.249* | -0.1  -1.4; 1.3 | *.936* | 1.0  -0.6; 2.6 | *.203* | 0.0  -1.8; 1.9 | *.958* |
| *Controlling for SES, 4 SDQ problem subscales score, age and sex | | | | | | | | | | |
